# Incorporating Circulating Plasma Interleukin-10 Enhanced Risk Predictability of Mortality in Acute Type A Aortic Dissection Surgery

**DOI:** 10.31083/RCM26334

**Published:** 2025-02-21

**Authors:** Yi-fei Diao, Zhi-bin Chen, Jia-xi Gu, Xin-yang Xu, Wen-feng Lin, Chun-ze Yuan, Jia-qi Xiong, Ming-hui Li, Bu-qing Ni, Sheng Zhao, Yong-feng Shao, Ying-yuan Zhang, Hong Liu

**Affiliations:** ^1^Department of Cardiovascular Surgery, First Affiliated Hospital of Nanjing Medical University, 210029 Nanjing, Jiangsu, China; ^2^Department of Cardiovascular Surgery, First Affiliated Hospital of Guangzhou Medical University, 510120 Guangzhou, Guangdong, China

**Keywords:** IL-10, LASSO, mortality, nomogram, type A aortic dissection

## Abstract

**Background:**

Acute type A aortic dissection (TAAD) is a life-threatening cardiovascular emergency with a high mortality rate. The peri-operative factors influencing in-hospital mortality among surgically treated TAAD patients remain unclear. This study aimed to identify key peri-operative risk factors associated with in-hospital mortality.

**Methods:**

Peri-operative laboratory data, surgical strategies, and TAAD-related risk factors, associated with mortality, were collected. Machine learning techniques were applied to evaluate the impact of various parameters on in-hospital mortality. Based on the findings, a nomogram model was developed and validated using area under the receiver operating characteristic curve (AUC) analysis, calibration plots, and internal validation methods.

**Results:**

A total of 199 patients with TAAD were included in the study cohort, which was divided into derivation and validation cohorts. Using the least absolute shrinkage and selection operator (LASSO) regression method, 66 features were narrowed down to six key predictors. These included age, lymphocyte count, use of continuous renal replacement therapy (CRRT), cardiopulmonary bypass (CPB) time, duration of mechanical ventilation, and postoperative interleukin-10 (IL-10) levels, all of which were identified as significant risk factors for in-hospital mortality following TAAD surgery.

**Conclusions:**

We developed and validated a predictive model, presented as a nomogram, to estimate in-hospital survival in patients with TAAD. Post-operative IL-10 was identified as an independent prognostic factor for patients with TAAD. The combination of IL-10 with five additional indicators significantly improved the predictive accuracy, demonstrating superiority over the use of any single variable alone.

**Clinical Trial Registration:**

This study protocol was registered at ClinicalTrials.gov (NCT04711889). https://clinicaltrials.gov/study/NCT04711889.

## 1. Introduction

Acute Stanford type A aortic dissection (TAAD) is a life-threatening condition characterized by high morbidity and mortality rates. Without surgical intervention, the mortality rate for TAAD patients increases by approximately 0.5% per hour during the initial 48 hours [1]. However, emergency surgical intervention reduces this rate significantly to 0.09% per hour within the same timeframe [1].

Given the life-threatening nature of TAAD, identifying peri-operative indicators to predict patient prognosis is crucial for optimizing post-operative care and improving early clinical decision-making. Using data from the German Registry for Acute Type A Aortic Dissection (GERAADA), researchers developed a scoring system to estimate 30-day mortality based on easily accessible parameters, including age, catecholamine use, and preoperative resuscitation [2]. Similarly, a 15-year German study identified additional risk factors, including prior cardiac surgery and blood transfusions [3]. However, these models are time-consuming and do not consider immune status.

Inflammation is a key contributor to the pathogenesis, progression, and prognosis of aortic dissection [4, 5]. Evidence also suggests that patients undergoing TAAD surgery may benefit from anti-inflammatory pharmacotherapy. A multicenter cohort study developed an inflammatory risk model to predict multiple organ dysfunction syndrome (MODS) following aortic replacement [6]. In this study, patients receiving personalized anti-inflammation treatment (e.g., intravenous Ulinastatin) following surgery showed a reduced risk of MODS. Based on these findings, we hypothesize that TAAD represents a heterogeneous inflammatory syndrome, and specific inflammatory markers may be utilized to categorize the disease severity.

Recent studies have highlighted the prognostic potential of inflammatory biomarkers in TAAD surgery, including interleukin-6 (IL-6) [7], C-reactive protein (CRP) [8], D-dimer [9], and the peripheral platelet-to-white blood cell ratio (PWR) [10]. Interleukin-10 (IL-10), an anti-inflammatory cytokine, plays an important role in modulating the human immune system, and it has been used to evaluate the long-term outcomes of solid tumors and non-solid tumors, such as hepatocellular carcinoma (HCC) [11], and non-Hodgkin’s lymphoma [12]. Previous research identified IL-10 as a biomarker for differentiating TAAD from other acute chest pain emergency, including acute myocardial infarction (AMI) and pulmonary embolism (PE) [13]. However, the prognostic value of circulating IL-10 in TAAD patients remains largely unexplored.

In this study, we recruited TAAD patients undergoing emergency surgery to evaluate the prognostic significance of baseline serum IL-10 levels on overall survival. Our findings aim to facilitate the early identification of high-risk patients and support personalized, precise treatment strategies to improve clinical outcomes.

## 2. Materials and Methods

### 2.1 Study Population

**Fig. 1.**
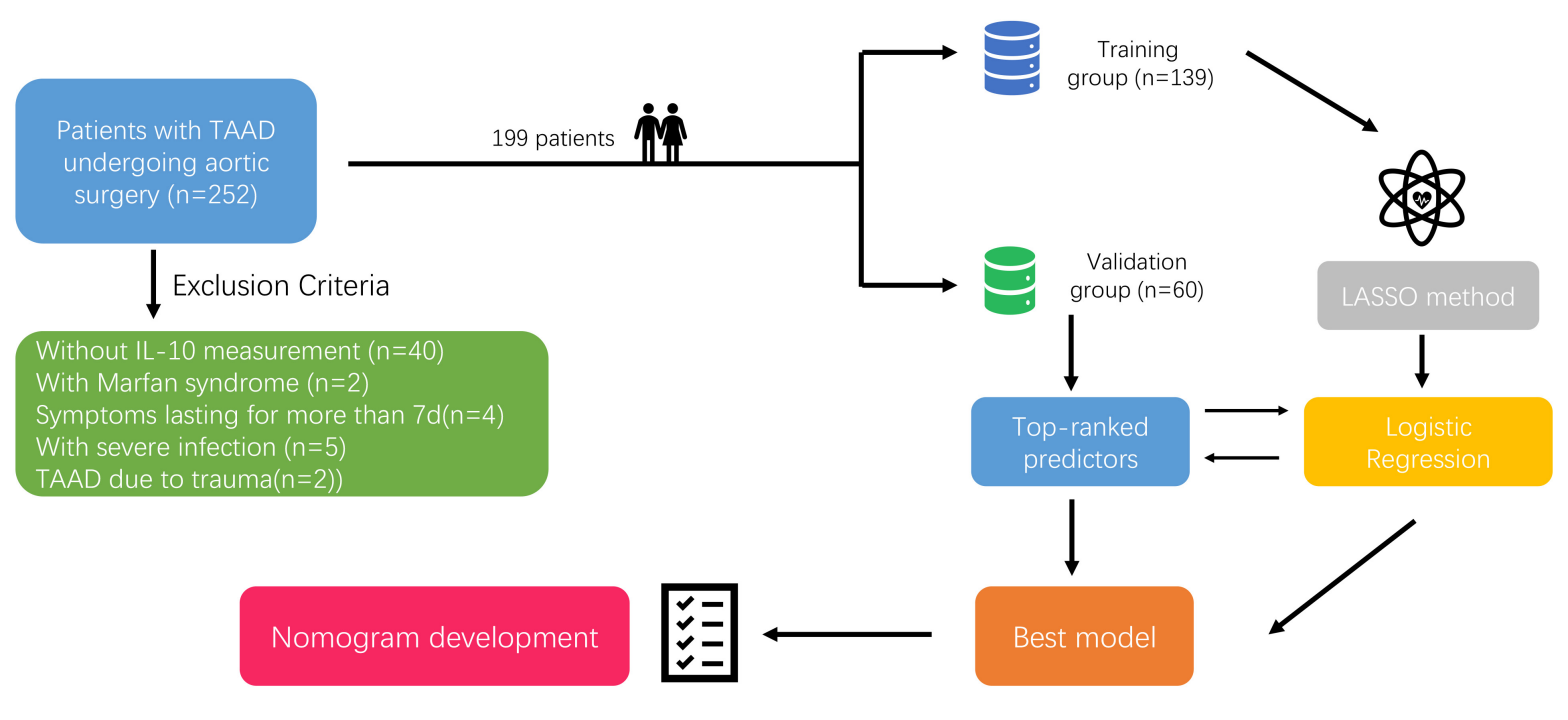
**Flowing chart for this study**. TAAD, type A aortic dissection; IL-10, interleukin-10; LASSO, least absolute shrinkage and selection operator.

We recruited TAAD patients (n = 252) undergoing emergency surgery at the First Affiliated Hospital of Nanjing Medical University and the First Affiliated Hospital of Guangzhou Medical University between July 2022 and February 2024. The inclusion criteria were: (a) diagnosed as TAAD according to aortic computed tomography angiography (CTA) and received emergency operation; (b) aged ≥18 years. The exclusion criteria were as follows: (a) no post-operative IL-10 measurement; (b) simultaneous diagnosis with Marfan syndrome (MFS); (c) symptoms exceeding 7 days; (d) patients with severe infection (Fig. 1). A total of 199 patients met the criteria and were included in the study.

### 2.2 Data Collection and Definition

Peripheral blood was extracted from all patients upon admission and following the operation (within 24 h of admission and within 6 h following the operation). Blood samples were immediately centrifuged, then the plasma was kept at –80 °C until analysis. Plasma interleukin levels were measured in duplicate using a standard enzyme-linked immune-absorbent assay (ELISA) kit. The database included demographic data, medical history, pre- & post-operative laboratory results and operative surgical related factors. After the hospitalization of TAAD patients, the deaths were recorded and the in-hospital mortality was calculated. The levels of glucose, lactic acid, base excess (BE), pH, partial pressure of carbon dioxide (PCO_2_), and HCO3- were measured through arterial blood gas analysis when patients are brought into the operating room. Other preoperative laboratory results were obtained upon patient admission. Total arch replacement was defined as the replacement of the entire aortic arch, including innominate artery (IA), left carotid artery (LCA), and left subclavian artery (LSA). Bentall procedure was defined as the replacement of the aortic valve, aortic root, and ascending aorta with a composite graft. The coronary arteries were then re-implanted into the graft. All surgeries were performed by the chief of surgeons, with over 10 years of experience in this field. The primary outcome was in-hospital mortality, defined as any death, regardless of cause after surgery, in this current hospitalization subsequent to the operation.

### 2.3 Statistical Analysis

Continuous variables were reported with mean ± standard deviation (SD) and were compared using Student’s independent t-test. Whereas median (interquartile range, IQR) and Mann-Whitney U test were used when the variables did not satisfy the normal distribution (Kolmogorov-Smirnov test). Categorial variables were presented as number (percentages, %), and were compared using Chi-square or Fisher’s exact test. Serum levels of IL-10 were presented in a dichotomized manner (i.e., high IL-10 group vs low IL-10 group). The cut-off values for age, cardiopulmonary bypass (CPB) time, lymphocyte and other continuous variables are based on Yuden index (the maximum vertical distance between receiver operating characteristic [ROC] curve and diagonal line; J = sensitivity + specificity – 1). The associations among these indicators were calculated through Spearman’s rank correlation (rho) and visualized by Heatmap.

The patient cohort was randomly divided into a training set (N1 = 139; 70%) and a test set (N2 = 60; 30%). In the training cohort, the clinical factors associated with in-hospital mortality were initially analyzed using the least absolute shrinkage and selection operator (LASSO) method [14, 15], which is suitable for the regression of high-dimensional data (This was due to the relatively large number of indices when compared to the number of subjects). A total of 66 variables were finally selected based on previous similar studies and clinical experience, and were standardized (standardize each predictor to have a mean of 0 and a standard deviation of 1). Regularization parameter (λ) was selected by 10-fold cross-validation. This parameter controls the strength of the penalty, as a larger λ increases bias but decreases variance, while a smaller λ does the opposite. For each fold, we fit the LASSO model with the current λ and calculated the mean squared error (MSE) on the validation set. Based on an appropriate λ (which yields the lowest MSE), 11 out of 66 factors were selected. In our analysis, the boundary conditions were set primarily through the selection of the λ parameter using cross-validation and the standardization of predictors. Next, multivariate logistic regression analysis was employed to examine the correlation between these variables and in-hospital mortality, with estimated odds ratios (OR) utilized for analysis [16]. The final model containing 6 variables was converted into a nomogram, and was tested in the validation cohort. The area under the receiver operating characteristic curve (AUC) was measured to quantify the discrimination performance of the model. Based on the cohort, we conducted decision curve analyses to determine the clinical usefulness of the model in both training and validation cohorts. This model’s calibration was assessed using calibration plots. SPSS Statistics (version 26, IBM, Armonk, NY, USA), GraphPad Prism (version 9.0, Dotmatics, Boston, MA, USA) and R software (version 4.3.0, Posit Software, Boston, MA, USA) were used to complete above statistical analysis. Statistical tests with *p* < 0.05 were considered significant.

## 3. Results

### 3.1 Baseline Characteristics

A total of 199 patients with TAAD were included in this study. The baseline patient characteristics are summarized in Table 1. The mean age was 53 years, with a mean body mass index (BMI) of 26.0 kg/m^2^, and 77% of the cohort were male. Patients who died or experienced prolonged hospital stays had significantly higher levels of IL-10 (118.9 pg/mL) compared to those who survived or had shorter stays (14.6 pg/mL) (*p* < 0.001).

**Table 1.** **Baseline demographic and clinical characteristics of the patient population**.

Variables			Total (n = 199)	Alive (n = 179)	Dead (n = 20)	*p*
Demographics						
	Gender, n (%)					0.26
		Male	153 (77)	140 (78)	13 (65)	
		Female	46 (23)	39 (22)	7 (35)	
	Hypertension, n (%)		146 (73)	133 (74)	13 (65)	0.531
	Diabetes, n (%)		7 (4)	6 (3)	1 (5)	0.529
	Age, Median (Q1,Q3)		53 (43, 61)	52 (42, 59.5)	62.5 (56.8, 68.3)	0.002
	BMI, Median (Q1,Q3)		26.0 (23.89, 28.7)	26.12 (23.95, 29)	24.2 (21.6, 26.4)	0.034
Laboratory results						
	WBC, Median (Q1,Q3)		12.2 (9.6, 14.6)	12.3 (9.7, 14.6)	12.0 (9.2, 14.4)	0.976
	Lym, Median (Q1,Q3)		0.96 (0.68, 1.33)	1.01 (0.69, 1.4)	0.84 (0.52, 0.98)	0.054
	Mono, Median (Q1,Q3)		0.62 (0.44, 0.9)	0.62 (0.44, 0.91)	0.58 (0.44, 0.84)	0.92
	Neu, Median (Q1,Q3)		10.3 (8.0, 12.5)	10.24 (8.0, 12.5)	10.27 (8.0, 12.0)	0.893
	Hb, Mean ± SD		133.44 ± 16.85	134.0 ± 16.91	127.8 ± 15.53	0.102
	MCV, Median (Q1,Q3)		92 (88.8, 94.3)	92 (88.8, 94.25)	92.5 (89.0, 95.0)	0.647
	RDW, Median (Q1,Q3)		13 (12.5, 13.43)	13 (12.5, 13.4)	13.1 (12.7, 13.55)	0.27
	PLT, Median (Q1,Q3)		164 (133, 205)	168 (136, 205)	136 (104, 186)	0.052
	PDW, Median (Q1,Q3)		14.2 (11.2, 16.3)	14.2 (11.1, 16.3)	14.9 (11.9, 16.2)	0.737
	Cr, Median (Q1,Q3)		77.5 (61, 110.5)	77.6 (61, 108.2)	74.4 (60.5, 113)	0.989
	BUN, Median (Q1,Q3)		6.89 (5.66, 8.54)	6.85 (5.64, 8.68)	7.37 (5.88, 7.97)	0.688
	AST, Median (Q1,Q3)		28.5 (23.3, 40.5)	27.7 (23.05, 39.7)	38.45 (27.05, 75.6)	0.012
	ALT, Median (Q1,Q3)		26.6 (19.5, 39.2)	27.6 (19.85, 38.85)	23.5 (16.45, 41.19)	0.65
	PT, Median (Q1,Q3)		12.2 (11.65, 13)	12.2 (11.6, 13)	12.35 (12.07, 13.1)	0.235
	INR, Median (Q1,Q3)		1.07 (1.02, 1.14)	1.07 (1.01, 1.14)	1.08 (1.05, 1.14)	0.4
	FIB, Median (Q1,Q3)		2.18 (1.62, 2.96)	2.2 (1.67, 2.99)	1.93 (1.38, 2.44)	0.077
	TT, Median (Q1,Q3)		17.4 (16.4, 18.5)	17.4 (16.3, 18.45)	17.75 (17.08, 21.4)	0.134
	D-dimer, Median (Q1,Q3)		5.57 (2.76, 9.81)	5.24 (2.68, 9.23)	7.72 (4.17, 10)	0.181
	cTnI, Median (Q1,Q3)		0.1 (0.1, 0.12)	0.1 (0.1, 0.1)	0.1 (0.1, 2)	0.255
	cTnT, Median (Q1,Q3)		14.76 (8.03, 50.3)	14.61 (7.72, 43.94)	39.03 (12.0, 116.2)	0.018
	CK-MB, Median (Q1,Q3)		1.65 (0.94, 4.62)	1.49 (0.9, 4.1)	5.58 (2.54, 33.64)	<0.001
	MYO, Median (Q1,Q3)		14.02 (9.63, 48.6)	13.31 (9.52, 34.2)	25.49 (12.8, 161.7)	0.018
	PH, Median (Q1,Q3)		7.35 (7.31, 7.39)	7.35 (7.32, 7.39)	7.32 (7.27, 7.37)	0.036
	PCO_2_, Median (Q1,Q3)		47 (41, 51)	47 (41, 51)	48.22 (41, 50.5)	0.6
	GLU, Median (Q1,Q3)		7.4 (6.45, 8.5)	7.3 (6.4, 8.43)	8.41 (6.88, 10.87)	0.05
	HCO3-, Median (Q1,Q3)		25.4 (23.35, 27.15)	25.4 (23.6, 27.2)	24 (21.53, 25.88)	0.06
	BE, Median (Q1,Q3)		0.4 (–2.1, 2.55)	0.47 (–1.85, 2.7)	–1.7 (–3.78, 0.56)	0.027
	LAC, Median (Q1,Q3)		1.8 (1.1, 2.75)	1.7 (1, 2.6)	2.3 (1.62, 3.15)	0.051
	Ccr, Median (Q1,Q3)		9.85 (7.2, 12.76)	10.1 (7.39, 12.85)	7.57 (5.73, 10.79)	0.038
	pWBC, Mean ± SD		12.49 ± 4	12.46 ± 3.97	12.76 ± 4.38	0.779
	pLym, Median (Q1,Q3)		0.58 (0.41, 0.85)	0.58 (0.41, 0.84)	0.56 (0.39, 0.88)	0.863
	pMono, Median (Q1,Q3)		0.88 (0.63, 1.17)	0.88 (0.61, 1.16)	1 (0.77, 1.21)	0.433
	pNeu, Mean ± SD		10.82 ± 3.58	10.81 ± 3.59	10.91 ± 3.63	0.91
	pHb, Median (Q1,Q3)		107 (100, 115)	107 (100, 114)	106.5 (100.8, 117)	0.765
	pPLT, Median (Q1,Q3)		112 (95, 140)	116 (97, 141)	94 (83, 125.5)	0.052
	pCr, Median (Q1,Q3)		99.1 (75.3, 130.8)	98.9 (76.1, 130.1)	103.9 (70.7, 142.7)	0.987
	pBUN, Median (Q1,Q3)		8.68 (7.3, 10.57)	8.68 (7.29, 10.49)	8.65 (8.09, 12.17)	0.385
	pALT, Median (Q1,Q3)		32 (24.15, 44.55)	31.9 (23.85, 44.1)	34.2 (25.53, 63.75)	0.416
	pCcr, Median (Q1,Q3)		7.94 (5.94, 9.68)	8.19 (6.03, 9.74)	7.05 (4.49, 8.47)	0.036
	IL-2, Median (Q1,Q3)		0.27 (0.01, 1.11)	0.32 (0.01, 1.13)	0.01 (0.01, 0.66)	0.408
	IL-4, Median (Q1,Q3)		0.3 (0.01, 0.96)	0.31 (0.01, 0.96)	0.01 (0.01, 0.7)	0.387
	IL-6, Median (Q1,Q3)		136.7 (67.8, 246.1)	135.7 (61.6, 231.3)	186.2 (109, 532.4)	0.01
	IL-10, Median (Q1,Q3)		20.24 (5.67, 69.62)	14.59 (5.02, 43.56)	118.89 (31, 357.6)	<0.001
	IFN-γ, Median (Q1,Q3)		1 (0.04, 2.9)	0.81 (0.04, 2.87)	1.94 (0.18, 3.35)	0.39
During Surgery						
	CA time, Median (Q1,Q3)		21 (16, 27)	21 (15.5, 27)	23 (17, 27)	0.271
	ACCT time, Median (Q1,Q3)		142 (117, 179.5)	140 (115, 172.5)	189.5 (148.8, 212)	0.003
	CPB time, Median (Q1,Q3)		190 (167.5, 231)	188 (165, 223.5)	231 (183, 253.5)	0.024
	Bentall, n (%)		35 (18)	28 (16)	7 (35)	0.056
	Total arch, n (%)		168 (84)	153 (85)	15 (75)	0.208
	RBC-trans, Median (Q1, Q3)		2.0 (0, 4)	1.5 (0, 4)	3.25 (2, 4)	0.002
	Plasma-trans, Median (Q1, Q3)		0 (0, 300)	0 (0, 287.5)	300 (0, 400)	0.007
	PLT-trans, Median (Q1, Q3)		10 (10, 10)	10 (0, 10)	10 (10, 20)	0.593
	Cryo-trans, Median (Q1, Q3)		9.7 (9, 10)	9.75 (9, 10)	9.5 (9, 10)	0.717
	Total-trans, Median (Q1, Q3)		1125 (750, 1775)	1100 (750, 1662.5)	1830 (1357.5, 2125)	<0.001
Peri-operative outcomes						
	The use of CRRT, n (%)		41 (21)	26 (15)	15 (75)	<0.001
	ICU-day, Median (Q1,Q3)		7 (4, 14)	7 (4, 13)	12.5 (4, 21.5)	0.169
	Hosp, Median (Q1,Q3)		17 (13.5, 25)	18 (14, 25)	13 (4, 23.5)	0.008
	Ventilation, Median (Q1,Q3)		36.13 (15, 123)	32.9 (14.36, 108.5)	118.9 (86.7, 334.1)	<0.001
	Any of malperfusion, n (%)		79 (40)	66 (37)	13 (65)	0.028
	Intestinal		21 (11)	15 (8)	6 (30)	0.01
	Limb		30 (15)	21 (12)	9 (45)	<0.001
	Cerebral		19 (11)	16 (9)	3 (15)	0.415
	Coronary		36 (18)	29 (16)	7 (35)	0.06

Q1, Q3: interquartile range; p-stands for postoperative: i.e., pWBC: post-operative white blood cell, 10^9^/L; -trans stands for blood transfusion, i.e., plasma-trans: transfusion of plasma, mL. SD, standard deviation; BMI, body mass index, kg/m^2^; WBC, white blood cell count, 10^9^/L; Lym, lymphocyte count, 10^9^/L; Mono, monocyte count, 10^9^/L; Neu, neutrophil count, 10^9^/L; Hb, hemoglobin, g/L; MCV, mean corpuscular volume; RDW, red cell distribution width; PDW, platelet distribution width; PLT, platelet count, 10^9^/L; Cr, creatinine, µmol/L; BUN, blood urea nitrogen, U/L; AST, aspartic transaminase, U/L; ALT, alanine aminotransferase, U/L; PT, prothrombin time, second; INR, international normalized ratio; FIB, fibrinogen, g/L; TT, thrombin time, second; cTnI, cardiac troponin-I, ng/L; cTnT, cardiac troponin-T, ng/L; CK-MB, creatine kinase-MB, ng/mL; MYO, myoglobin, ng/mL; BE, base excess, mmol/L;PH, potential of hydrogen; PCO_2_, partial pressure of carbon dioxide, mmHg; HCO3-, bicarbonate ion, mmol/L; GLU, glucose, µmol/L; LAC, lactic acid, µmol/L; Ccr, creatinine clearance rate, based on Cockcroft-Gault formula, mL/min; IL, interleukin, pg/mL; IFN-γ, interferon-γ, pg/mL; CA, circulation arrest time, minute; ACCT, aortic cross-clamping time, minute; CPB, cardiopulmonary bypass time, minute; Total arch, total arch replacement using a vascular graft in combination with implantation of a special stented graft into the descending aorta; Bentall procedure was defined as the replacement of the aortic valve, aortic root, and ascending aorta with a composite graft. The coronary arteries were then re-implanted into the graft; RBC, red blood cell, U; Cryo, cryoprecipitate, U; Total-trans, total administration of all blood and blood products, mL; CRRT, continuous renal replacement therapy; Hosp, length of hospital stay, day; ICU, intensive care unit; Ventilation, duration of mechanical ventilation, hours.

The median IL-10 level was 20.2 pg/mL, ranging from 0.86 to 492.18 pg/mL. This median value was used as the cut-off, categorizing cytokine levels above 20.2 pg/mL as high. Patients in the high-IL-10 group exhibited significantly higher mortality compared to those in the low-IL-10 group (24% vs. 5.1%, *p* < 0.001). Kaplan-Meier analysis revealed improved in-hospital survival rates for patients with admission IL-10 levels ≤20.2 pg/mL (log-rank *p* = 0.003), consistent with the aforementioned findings (**Supplementary Fig. 1A**). Similarly, patients with higher IL-10 levels had prolonged intensive care unit (ICU) stays before transitioning to the general wards (log-rank *p* = 0.006) (**Supplementary Fig. 1B,C**).

Correlation analysis further demonstrated a significant association between IL-10 levels and the incidence of post-operative end-organ malperfusion (rho = 0.558, *p* < 0.001) (**Supplementary Fig. 2**), whereas IL-6 (rho = 0.232, *p* = 0.473) showed no clear correlation. Elevated IL-10 levels were particularly observed in patients with intestinal, limb, brain, or coronary artery malperfusion.

Next, patients were divided into the training cohort (70%) and validation cohort (30%) stochastically. Their baseline characteristics were summarized in **Supplementary Table 1**.

### 3.2 Feature Selection

**Fig. 2.**
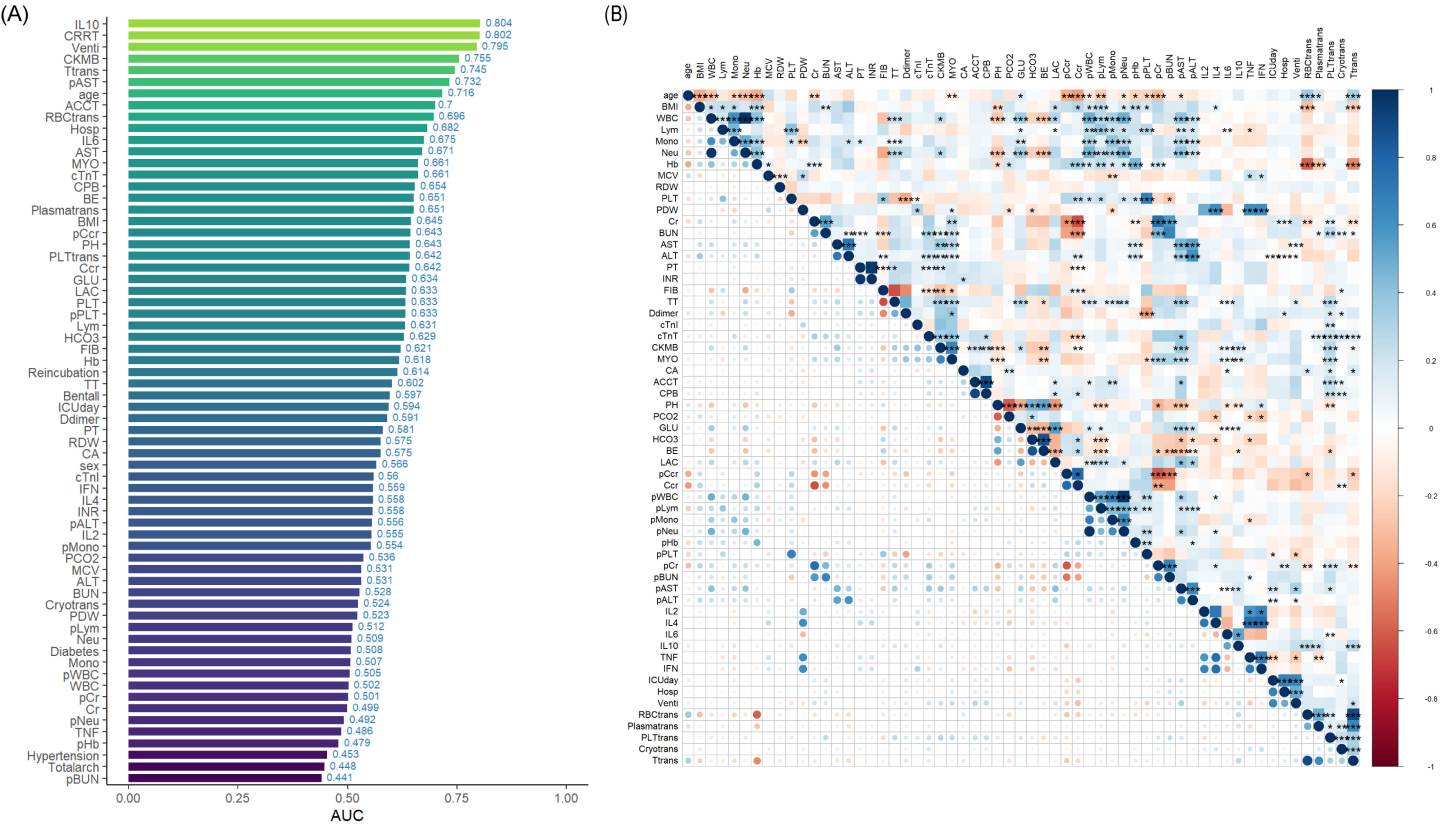
**Performance and correlation analysis of prognostic indicators**. (A) Areas under the ROC curves (AUC) for all 66 predictors ranked in descending order. (B) Heatmap illustrating correlations among the predictors. The color spectrum in the heatmap varies from deep blue, indicating positive correlations, to deep red, indicating negative correlations. Statistical significance is denoted as follows: * *p* < 0.05, ** for *p* < 0.01, and *** for *p* < 0.001. ROC, receiver operating characteristic curve; Venti, time of mechanical ventilation; Ttrans, total administration of all blood and blood products; pAST, postoperative aspartic transaminase.

We calculated the AUC for all 66 indicators and ranked them in ascending order (Fig. 2A). The top five indicators, based on AUC values, were IL-10 (0.804), use of continuous renal replacement therapy (continuous renal replacement therapy, CRRT; 0.802), duration of mechanical ventilation (0.795), serum creatine kinase-myocardial band (creatine kinase-MB, CK-MB; 0.755), and total blood transfusion (0.745). A matrix heatmap analysis was conducted to evaluate the correlation among each indicator (Fig. 2B).

**Fig. 3.**
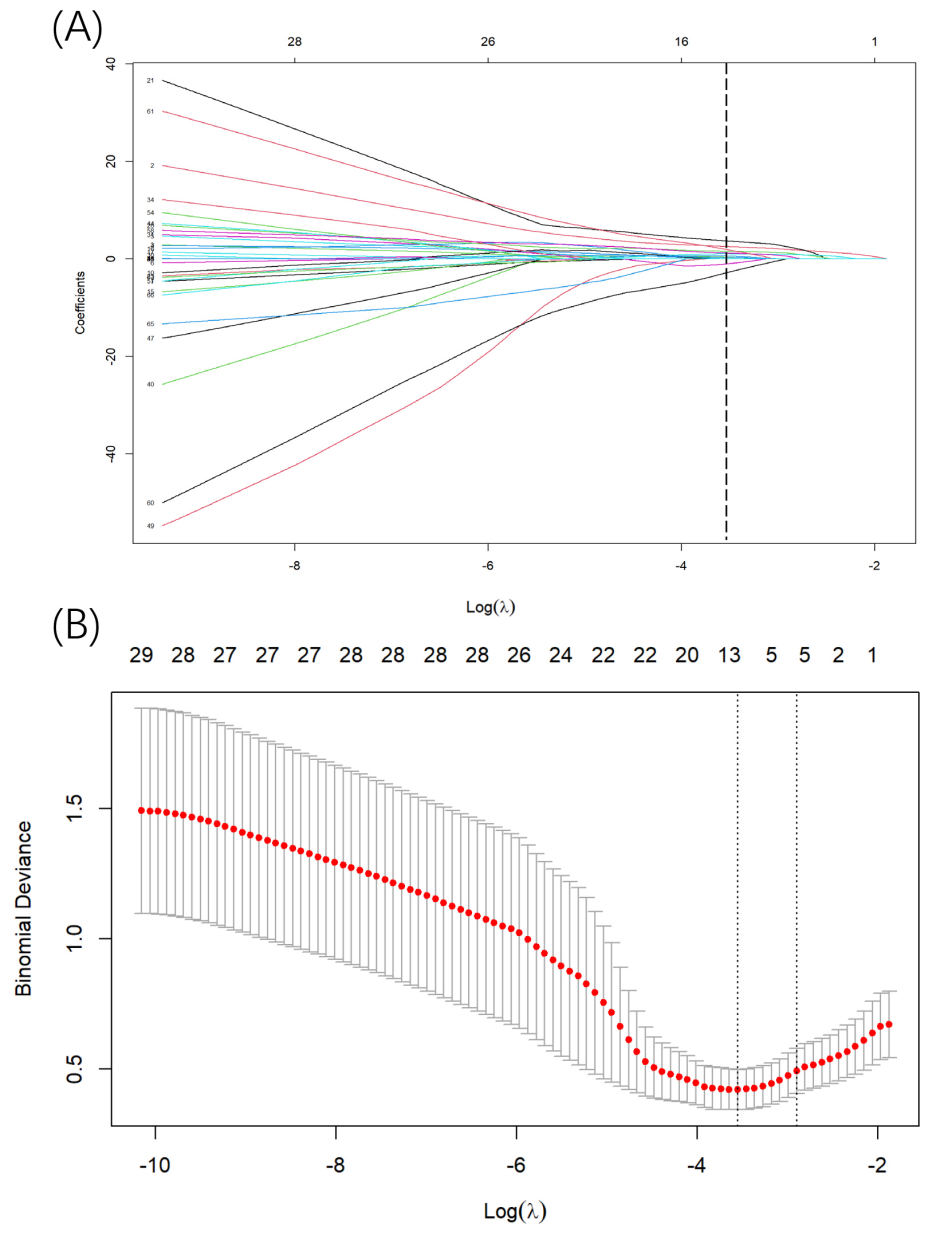
**Screening of variables based on Lasso regression**. (A) The variation characteristics of the coefficient of variables. As the value of λ decreased, the degree of model compression increased and the function of the model to select important variables increased. Dotted line was drawn at the value selected using 10-fold cross-validation, where optimal λ resulted in 11 non-zero coefficients. (B) The selection process of the optimum value of the parameter λ in the Lasso regression model by cross-validation method. The value in the middle of the two dotted lines is the range of the maximum and minimum values of log(λ).

The Lasso method was employed for parameter screening in the training cohort, and the variation characteristics of the coefficient of these variables were illustrated in Fig. 3A. The model demonstrated exceptional performance at the λ value corresponding to the lowest MSE (Fig. 3B). With final λ = 0.0457, the 66 indicators were reduced to 11 potential predictors.

The selected variables included age, lymphocyte count, blood urea nitrogen (BUN), aspartate aminotransferase (AST), thrombin time (TT), glucose, platelet transfusion, use of CRRT, CPB time, duration of mechanical ventilation, and postoperative IL-10 levels. Variables identified through the Lasso method were further subjected to multivariate logistic regression analysis to determine independent risk factors associated with mortality. Backward step-wise selection was applied. As summarized in Table 2, the independent risk factors for in-hospital mortality included: age ≥56 years (*p* = 0.0456), perioperative lymphocyte count ≥0.6 × 10^9^/L (*p* = 0.0034), CRRT use (*p* = 0.0311), CPB time ≥230 minutes (*p* = 0.0260), mechanical ventilation ≥62 hours (*p* = 0.0113), and postoperative IL-10 (*p* = 0.0105). The mortality risk among patients with age ≥56 was 5.29 times higher compared to younger patients, while each incremental increase in IL-10 levels raised the mortality risk by 1.71-fold compared to baseline.

**Table 2.** **Multivariable logistic regression analysis of possible predictors of in-hospital mortality for TAAD**.

		Crude Method				Adjust Method			
Variable		*p* value	OR	(95% CI)		*p* value	OR	(95% CI)	
Socio-demographics									
	Age ≥56 years	0.0431	76.269	1.143	5089	0.046	5.287	3.896	31.200
Blood tests									
	Lymphocyte ≥0.60	0.0549	126	0.904	1760	0.003	17.416	2.571	117.990
	BUN	0.0854	0.018	0.0002	1.750				
	AST	0.5859	0.721	0.223	2.335				
	TT	0.0639	0.007	0.0004	1.167				
	Glucose	0.228	0.451	0.04	124.9				
	Transfusion of platelet	0.729	5925	0.00	7845				
Time of CPB ≥230 min		0.0800	9.737	0.762	124	0.0260	6.889	1.260	37.667
Duration of mechanical ventilation ≥62 h		0.6173	4217	0.0000	11170	0.0113	31.258	2.176	448.99
	Use of CRRT	0.0725	22.335	0.753	662	0.0311	6.667	1.188	37.420
Marker									
	IL-10	0.0428	1.004	0.995	1.014	0.0105	1.711	1.134	2.582

OR, odds ratio; 95% CI, 95% of confidence interval; The Crude Method contained eleven variables selected by LASSO method, The Adjust method contained the final six variables.

### 3.3 Develop and Validate the Nomogram

**Fig. 4.**
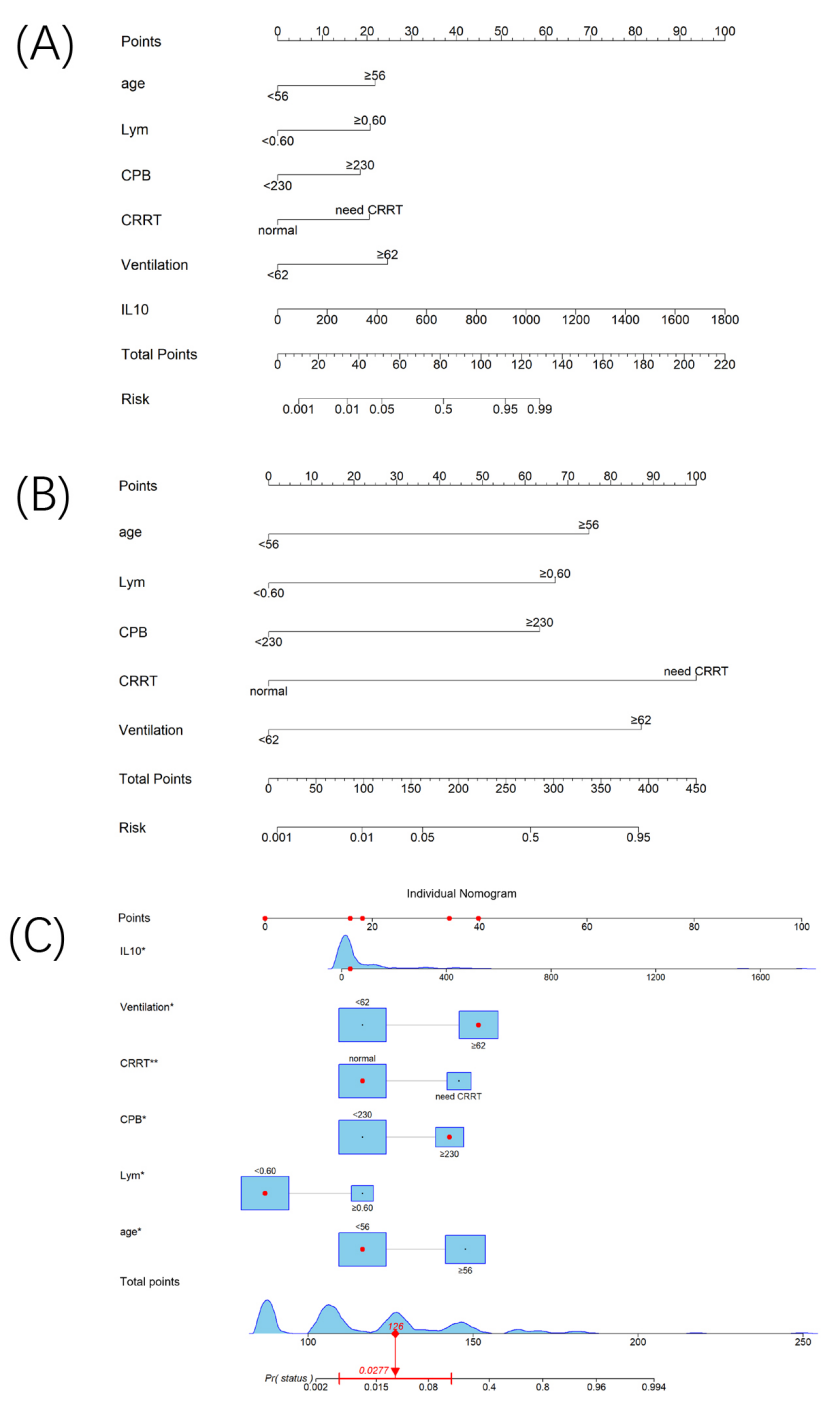
**Nomogram to predict mortality in patients with TAAD**. The nomogram was developed in the primary cohort, incorporating IL-10 levels, age, lymphocyte count, duration of CPB and mechanical ventilation, and CRRT status. (A) Nomogram based on the classic model incorporating IL-10. (B) Nomogram based on the classic model without IL-10. (C) Example application of the nomogram for an individual patient. Basic information of this case: 54-year-old male with a CPB duration of 239 minutes, IL-10 level of 34 pg/mL, lymphocyte count of 0.54 × 10^9^/L, and mechanical ventilation duration of 373 hours. The patient did not require CRRT during hospitalization. The cumulative risk score was 126 points, corresponding to an estimated mortality risk of 2.8%. This patient was discharged in good health 34 days post-surgery. Pr (status) refers to the predicted probability of death for an individual based on the model.

The six predictors identified through Lasso-Logistic regression analysis were used to construct a nomogram (Fig. 4A), which achieved a high C-index of 0.967, indicating excellent discriminative ability for predicting in-hospital mortality. In comparison, a base nomogram containing only conventional predictors (Fig. 4B) yielded a lower C-index of 0.905. This improvement demonstrates the added predictive value of incorporating the six novel predictors, particularly IL-10 levels, into the model. Fig. 4C illustrates a practical application of our proposed nomogram in estimating survival rates, showcasing its potential for personalized risk assessment and decision-making in clinical practice.

**Fig. 5.**
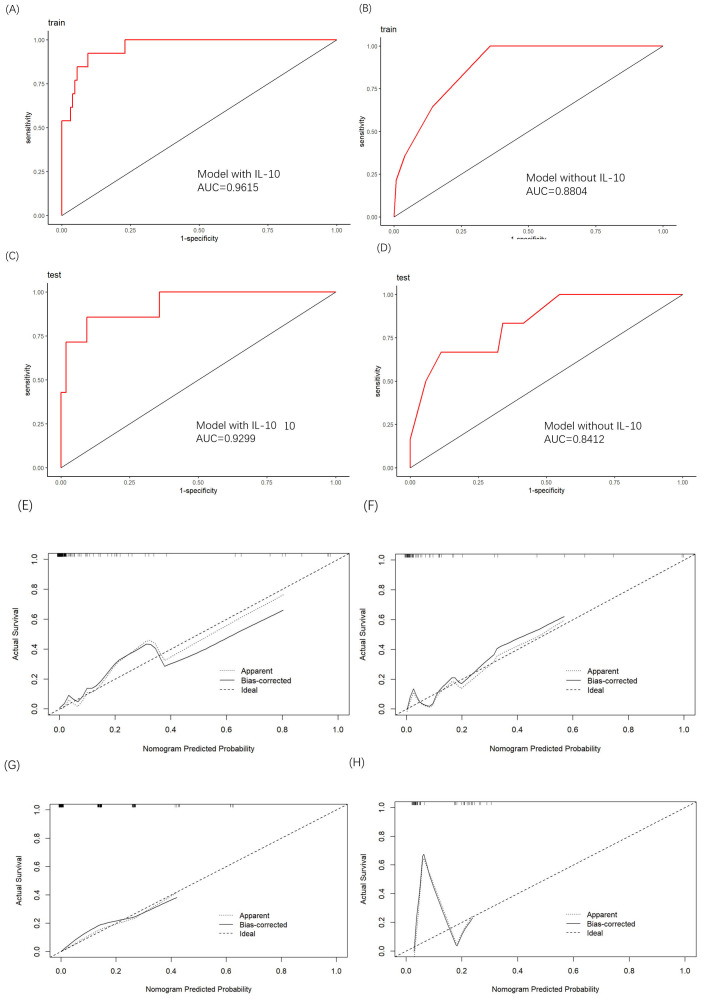
**Validation of the nomogram**. (A,B) ROC curves depicting the AUC of the inflammatory model and base model in the training cohort. (C,D) ROC curves depicting the AUC of the inflammatory model and base model in the test cohorts, respectively. (E,F) Calibration curves for the inflammatory model in the training and test cohorts, demonstrating alignment between predicted and observed mortality. (G,H) Calibration curves for the base model in the training and test cohorts, illustrating a notable discrepancy in performance between cohorts. In all calibration curves, the y-axis depicts the actual mortality rate, while the x-axis illustrates the predicted risk of death. The diagonal dotted line signifies perfect prediction by an ideal model, while the solid line represents the performance of the nomogram, with a closer alignment to the diagonal dotted line indicating superior predictive capability.

The ROC curve analysis demonstrated that the inflammatory nomogram outperformed the base nomogram in discrimination accuracy. In the training cohort, the inflammatory nomogram achieved an AUC of 0.962 compared to 0.880 for the base nomogram (Fig. 5A,B), while in the test cohort, the AUC was 0.930 versus. 0.884 (Fig. 5C,D), underscoring its superior predictive performance. Calibration was assessed using the Hosmer–Lemeshow test, which confirmed good calibration for both models. The inflammatory nomogram yielded a *p*-value of 0.626, while the base nomogram achieved a p-value of 0.438. The calibration curves (Fig. 5E,F) demonstrated strong agreement between the predicted and observed probabilities in the primary and validation cohort. However, the base model’s calibration performance showed inconsistencies, with a notable discrepancy between its performance in the training cohort (Fig. 5G) and the test cohort (Fig. 5H). These findings highlight the robustness and reliability of the inflammatory nomogram, particularly for clinical decision-making in diverse patient settings.

### 3.4 Clinical Use

**Fig. 6.**
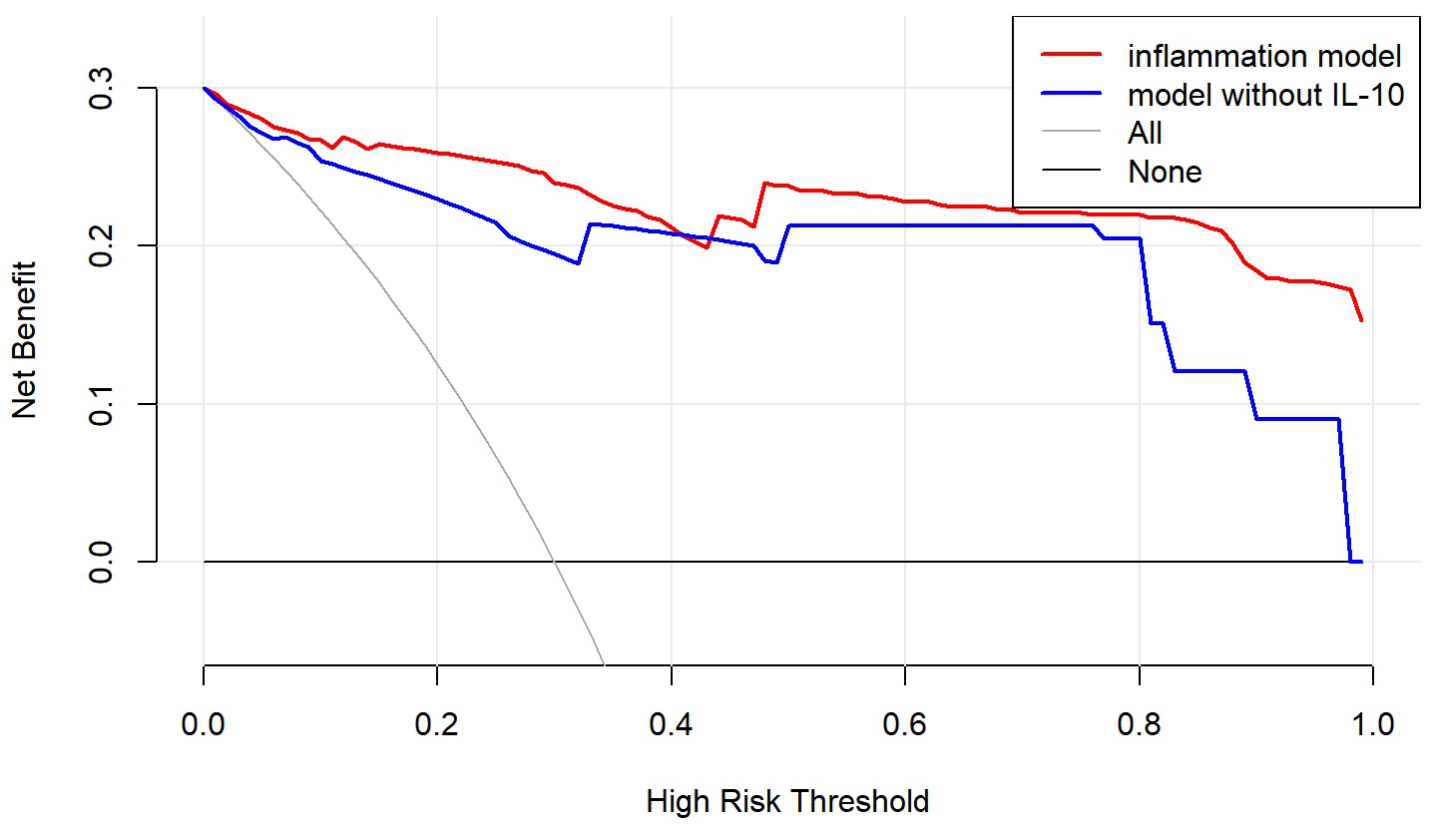
**Decision curve analysis for the nomogram models**. The y-axis measures the net benefit. The red line depicts the inflammatory nomogram, incorporating IL-10, applied to all 199 patients. The blue line represents the base nomogram, excluding IL-10. The thin black line assumes that all patients die following surgery, while the bold black line assumes that all patients survive. The net benefit was calculated by subtracting the proportion of false positive patients from the proportion of true positive patients, weighted by the relative harm of withholding necessary treatment versus providing unnecessary treatment.

The clinical utility of the nomogram was evaluated using decision curve analysis in both the training and validation cohorts (Fig. 6). This analysis demonstrated that the inflammatory nomogram consistently provided superior clinical benefits compared to the base nomogram. Specifically, within a risk threshold range of 45–95%, the inflammatory model resulted in a significantly higher net benefit for guiding treatment decisions. These findings highlight the potential of the inflammatory nomogram to enhance personalized treatment strategies by improving risk stratification and supporting more informed clinical decision-making.

## 4. Discussion

TAAD is a life-threatening cardiac emergency with significant clinical and public health implications. The incidence of TAAD has been reported as 11.9 cases per 100,000 patients per year in the Berlin-Brandenburg region and ranges from 5.93–24.92 cases per 100,000 inhabitants per year across different emergency departments [17, 18]. Currently, the only effective treatment for TAAD is emergency surgical repair. Numerous studies have attempted to identify risk factors associated with in-hospital mortality and post-operative complications in TAAD patient [8, 19]. In this study, we evaluated the prognostic significance of baseline serum IL-10 levels on overall survival in TAAD patients and established a straightforward nomogram for predicting in-hospital mortality.

Using LASSO methods and multivariate logistic regression, we identified six key predictors of in-hospital mortality in TAAD patients: age ≥56 years, perioperative lymphocyte ≥0.6 × 10^9^/L, use of CRRT, CPB time ≥230 minutes, mechanical ventilation time ≥62 hours and postoperative IL-10 levels. Based on these predictors, we developed a nomogram model that demonstrated significant utility as a prognostic tool. These six indicators reflect both the patients’ overall clinical condition and key inflammatory characteristics, underscoring the interplay between systemic inflammation and TAAD outcomes.

In this study, we observed for the first time that deceased individuals with TAAD exhibited significantly elevated serum IL-10 levels compared to survivors. Specifically, 120 out of 158 (76%) patients had IL-10 levels exceeding the upper limit of our hospital’s standard reference (4.91 pg/mL), suggesting that IL-10 may serve as a potential rule-in biomarker for TAAD. This finding is consistent with prior research suggesting that incorporating IL-10 into diagnostic panels alongside cardiac troponin T (cTnT) and D-dimer may improve the discrimination of TAAD from other acute chest pain conditions, such as AMI, and PE [13].

We demonstrated that serum IL-10 levels serve as an independent prognostic factor for TAAD patients after surgery. The in-hospital mortality rate of patients in the high IL-10 cohort was more than five times that of patients in the low IL-10 cohort (*p* < 0.001). To our knowledge, this is the first study to systematically evaluate the independent prognostic impact of the serum IL-10 levels in patients with TAAD.

The prognostic impact of IL-10 has also been observed in other conditions, such as unresectable hepatocellular carcinoma (HCC) and Coronavirus Disease 2019 (COVID-19) [11, 20]. Among patients with unresectable HCC, those with high IL-10 levels had significantly worse overall survival compared to those with low IL-10 levels (5.0 months vs 14.9 months). Similarly, COVID-19 patients with elevated IL-10 levels upon admission, were more likely develop severe disease. These studies highlight the strong correlation between elevated IL-10 levels and unfavorable clinical outcomes across a range of conditions, which is consistent with our own findings.

The mechanism underlying the association between elevated IL-10 levels and unfavorable outcomes remains unclear, as IL-10 is traditionally regarded as a classical anti-inflammatory cytokine [21]. In solid tumor, it has been hypothesized that IL-10 creates an immunosuppressive microenvironment conducive to tumor progression, potentially by interacting directly with cancer cells to promote cellular proliferation [22]. In vascular smooth muscle cells (VSMCs), previous studies have demonstrated that physiological doses of IL-10 inhibit proliferation and migration [23, 24]. Similarly, localized overexpression of IL-10 in cardiac allografts has been reported to significantly prolong allograft survival by inducing apoptosis of allogenic infiltrative CD8^+^ cells [25]. However, the impact of excessive IL-10 remains unclear. In individuals with systemic inflammatory disorders, the administration of high-dose IL-10 was found to paradoxically elicit pro-inflammatory effects by upregulating the production of other pro-inflammatory cytokines, such as IFN-γ [26]. These findings suggest that the role of IL-10 in inflammation is dose-dependent and context-specific. Future *in vivo* or *in vitro* studies should consider these factors into consideration to more accurately replicate the microenvironment of TAAD and clarify the role of IL-10 in its pathogenesis.

The prevailing consensus is that TAAD is characterized by the degradation of the extracellular matrix (ECM) and the phenotypic transformation of VSMCs, ultimately resulting in aortic aneurysms, dissection, and rupture [27, 28]. The tearing and damage of the aortic inner layer rapidly activates the coagulation and inflammation system, recruiting immune cells and triggering an inflammatory cascade in TAAD [28]. It is reported that IL-10 could be expressed by multiply immune cells, including T regulatory cells, monocytes, macrophages and dendritic cells, underscoring its pivotal role as a feedback regulator of diverse immune responses [29]. Hence, we hypothesize that elevated IL-10 levels in TAAD patients may represent an attempt to mitigate hyper-inflammation and limit tissue damage, although these efforts fail to suppress the inflammatory cascade. Supporting this hypothesis, a recent study demonstrated that following a peripheral immune insult, intestinal transient receptor potential ankyrin 1 (TRPA1)-positive vagal sensory neurons, which were activated by IL-10, would subsequently transmit signals to the brain and activate the brain-body axis [30]. This signaling loop ultimately enhances IL-10 expression, establishing a positive feedback mechanism critical for dampening inflammation. However, prolonged and excessive activation of this circuit significantly increased bacterial load in a model of bacterial infection [30]. These findings suggest a complex, context-dependent role for IL-10 and align with our hypothesis. Further studies are needed to elucidate the detailed pathological mechanisms underlying IL-10’s role in TAAD.

Identifying an optimal cut-off value for IL-10 is crucial for its effective use as a prognostic indicator in TAAD. In our study, the cut-off value was determined using the median IL-10 level across all 199 patients. Other cut-off values, computed through ROC curve analysis or the integral mean logarithmic value, failed to demonstrate statistical significance (*p* > 0.1) in the LASSO or logistic regression models. This limitation may be attributed to the relatively small sample size of our cohort. We propose that with a larger study population, a more refined categorization of IL-10 levels could enhance its utility as a risk indicator.

Some limitations existed in this study. (1) Study Design and Population: This was a retrospective study conducted on a cohort of only 199 Asian patients. As such, it is unclear whether the results could be generalized to Western populations or to patients with different etiologic factors. Further research involving a larger, more diverse cohort and extended post-operative follow-ups is currently underway. (2) Timing of IL-10 Measurement: IL-10 levels were measured after the operation. While pre-operative and sequential post-operative measurements might have provided greater insight, this approach was constrained by the time-intensive nature of IL-10 quantification via ELISA. Additionally, previous studies have demonstrated that the concentrations of these inflammatory markers, including IL-10, tend to persist post-surgery, regardless of dissection repair [31, 32]. To minimize the potential impact of operative factors, such as CPB time and hypothermic circulatory arrest (HCA) time, we opted not to conduct pre-operative testing for IL-10. Instead, we chose to perform a limited and standardized testing of IL-10 after the patients had returned from the operation room. (3) IL-10 Polymorphism: This study did not investigate the potential impact of *IL-10* genetic polymorphisms. A previous study suggested that specific polymorphisms, such as the single nucleotide ploymorphism (SNP) A/G–1082 variant in the *IL-10* promoter region, may be linked to an increased susceptibility to severe sepsis [33]. However, it is important to note that the primary objective of this study was to assess IL-10 as a marker with broad clinical applicability. Our clear and reproducible measurement of IL-10 underscores its potential utility in routine clinical practice.

## 5. Conclusions

In conclusion, serum IL-10 levels, when combined with other commonly used clinical parameters, may serve as a valuable prognostic tool for predicting the clinical progression of TAAD. Identifying high-risk patients immediately following emergency surgery using this model may facilitate timely interventions. For patients with higher score in our nomogram, early anti-inflammation therapy and personalized care should be prioritized. Future studies with a larger, more diverse cohort are necessary to validate the results of this two-center study. The levels of IL-10 and other immune markers, such as IL-6, CRP, and TNF-α should be analyzed at multiple time points before and after surgery to better understand their temporal dynamics and their roles in TAAD progression.

## Data Availability

The datasets analyzed during the current study are available from the corresponding author on a reasonable request. Further inquiries can be directed to the corresponding author. Codes for LASSO and Nomogram used in R in this paper can be found through “https://github.com/FFcaridac/IL-10-lasso.git”.

## Abbreviations

IL-10, interleukin-10; CPB, cardiopulmonary bypass; Lym, lymphocyte; TAAD, type A Aortic Dissection; CRRT, continuous renal replacement therapy; CT, computed tomography; ICU, intensive care unit; ALT, alanine transaminase; AST, aspartate transaminase.

## Supplementary Material

Supplementary material associated with this article can be found, in the online version, at https://doi.org/10.31083/RCM26334.
