# Supplementary material for: Incorporating Circulating Plasma Interleukin-10 Enhanced Risk Predictability of Mortality in Acute Type A Aortic Dissection Surgery
Source: Rev Cardiovasc Med. 2025 Feb 21;26(2):26334. doi: 10.31083/RCM26334 (PMC11868896; doi:10.31083/RCM26334)
Supplement: Supplementary file 1 [file 2153-8174-26-2-26334-s1.docx]

|  | Train group(n=140) | | | | Validation group(n=59) | | | |
| --- | --- | --- | --- | --- | --- | --- | --- | --- |
| Variables | **Total** | **alive(n= 126)** | **dead(n=14)** | ***p*** | **Total** | **alive(n = 53)** | **dead(n = 6)** | ***p*** |
| sex, n (%) |  |  |  | 0.74 |  |  |  | 0.114 |
| male | 107 (76) | 97 (77) | 10 (71) |  | 46 (78) | 43 (81) | 3 (50) |  |
| female | 33 (24) | 29 (23) | 4 (29) |  | 13 (22) | 10 (19) | 3 (50) |  |
| Hypertension, n (%) | 104 (74) | 96 (76) | 8 (57) | 0.193 | 42 (71) | 37 (70) | 5 (83) | 0.662 |
| Diabetes, n (%) | 6 (4) | 6 (5) | 0 (0) | 1 | 1 (2) | 0 (0) | 1 (17) | 0.102 |
| age, n (%) |  |  |  | 0.01 |  |  |  | 0.023 |
| <56 | 80 (57) | 77 (61) | 3 (21) |  | 37 (63) | 36 (68) | 1 (17) |  |
| ≥56 | 60 (43) | 49 (39) | 11 (79) |  | 22 (37) | 17 (32) | 5 (83) |  |
| BMI, n (%) |  |  |  | 0.012 |  |  |  | 1 |
| <24.5 | 52 (37) | 42 (33) | 10 (71) |  | 17 (29) | 15 (28) | 2 (33) |  |
| ≥24.5 | 88 (63) | 84 (67) | 4 (29) |  | 42 (71) | 38 (72) | 4 (67) |  |
| WBC, n (%) |  |  |  | 0.527 |  |  |  | 1 |
| <13.3 | 84 (60) | 74 (59) | 10 (71) |  | 36 (61) | 32 (60) | 4 (67) |  |
| ≥13.3 | 56 (40) | 52 (41) | 4 (29) |  | 23 (39) | 21 (40) | 2 (33) |  |
| Lymphocyte, n (%) |  |  |  | 0.055 |  |  |  | 0.013 |
| <0.60 | 117 (84) | 108 (86) | 9 (64) |  | 47 (80) | 45 (85) | 2 (33) |  |
| ≥0.60 | 23 (16) | 18 (14) | 5 (36) |  | 12 (20) | 8 (15) | 4 (67) |  |
| IL-6, n (%) |  |  |  | 0.002 |  |  |  | 0.076 |
| <403.7 | 117 (84) | 110 (87) | 7 (50) |  | 54 (92) | 50 (94) | 4 (67) |  |
| ≥403.7 | 23 (16) | 16 (13) | 7 (50) |  | 5 (8) | 3 (6) | 2 (33) |  |
| IL-10, n (%) |  |  |  | 0.002 |  |  |  | 0.008 |
| <15.1 | 60 (43) | 60 (48) | 0 (0) |  | 31 (53) | 31 (58) | 0 (0) |  |
| ≥15.1 | 80 (57) | 66 (52) | 14 (100) |  | 28 (47) | 22 (42) | 6 (100) |  |
| Ventilation, n (%) |  |  |  | 0.001 |  |  |  | 0.001 |
| <62 hrs | 81 (58) | 79 (63) | 2 (14) |  | 38 (64) | 38 (72) | 0 (0) |  |
| ≥62 hrs | 59 (42) | 47 (37) | 12 (86) |  | 21 (36) | 15 (28) | 6 (100) |  |
| T-transfusion, n (%) |  |  |  | 0.005 |  |  |  | 0.008 |
| <1500ml | 75 (54) | 73 (58) | 2 (14) |  | 31 (53) | 31 (58) | 0 (0) |  |
| ≥1500ml | 65 (46) | 53 (42) | 12 (86) |  | 28 (47) | 22 (42) | 6 (100) |  |
| ACCT, n (%) |  |  |  | 0.014 |  |  |  | 0.064 |
| <164min | 94 (67) | 89 (71) | 5 (36) |  | 41 (69) | 39 (74) | 2 (33) |  |
| ≥164 min | 46 (33) | 37 (29) | 9 (64) |  | 18 (31) | 14 (26) | 4 (67) |  |
| CPB, n (%) |  |  |  | 0.023 |  |  |  | 0.092 |
| <230min | 101 (72) | 95 (75) | 6 (43) |  | 47 (80) | 44 (83) | 3 (50) |  |
| ≥230min | 39 (28) | 31 (25) | 8 (57) |  | 12 (20) | 9 (17) | 3 (50) |  |
| BE, n (%) |  |  |  | 0.071 |  |  |  | 0.386 |
| <-0.7 | 47 (34) | 39 (31) | 8 (57) |  | 25 (42) | 21 (40) | 4 (67) |  |
| ≥-0.7 | 93 (66) | 87 (69) | 6 (43) |  | 34 (58) | 32 (60) | 2 (33) |  |
| LAC, n (%) |  |  |  | 0.075 |  |  |  | 0.656 |
| <1.35 | 56 (40) | 54 (43) | 2 (14) |  | 18 (31) | 17 (32) | 1 (17) |  |
| ≥1.35 | 84 (60) | 72 (57) | 12 (86) |  | 41 (69) | 36 (68) | 5 (83) |  |
| pCcr, n (%) |  |  |  | 0.155 |  |  |  | 0.08 |
| <8.69 | 80 (57) | 69 (55) | 11 (79) |  | 38 (64) | 32 (60) | 6 (100) |  |
| ≥8.69 | 60 (43) | 57 (45) | 3 (21) |  | 21 (36) | 21 (40) | 0 (0) |  |
| Ccr, n (%) |  |  |  | 0.249 |  |  |  | 0.036 |
| <8.56 | 55 (39) | 47 (37) | 8 (57) |  | 24 (41) | 19 (36) | 5 (83) |  |
| ≥8.56 | 85 (61) | 79 (63) | 6 (43) |  | 35 (59) | 34 (64) | 1 (17) |  |
| IL-2, Median (Q1,Q3) | 0.31 (0.01, 1.1) | 0.4 (0.01, 1.13) | 0.01 (0.01, 0.57) | 0.145 | 0.21 (0.01, 1.08) | 0.21 (0.01, 1.02) | 0.2 (0.01, 2.86) | 0.549 |
| IL-4, Median (Q1,Q3) | 0.3 (0.01, 0.96) | 0.3 (0.01, 0.96) | 0.01 (0.01, 0.92) | 0.605 | 0.26 (0.01, 0.96) | 0.46 (0.01, 1) | 0.03 (0.01, 0.34) | 0.453 |
| IL-6, Median (Q1,Q3) | 145.6 (72.23, 263.14) | 140.33 (68.04, 235.08) | 301.48 (125.63, 444.41) | 0.013 | 123.09 (58.59, 228.12) | 123.09 (57.3, 223.09) | 121.5 (72.38, 878.92) | 0.347 |
| IL-10, Median (Q1,Q3) | 21.74 (5.88, 62.48) | 18.52 (5.23, 43.51) | 118.89 (38.27, 300.04) | < 0.001 | 11.34 (4.97, 72.79) | 9.51 (4.56, 43.28) | 119.06 (38.97, 1184.51) | 0.014 |
| IFN, Median (Q1,Q3) | 0.7 (0.05, 3.24) | 0.6 (0.04, 2.89) | 2.38 (0.1, 3.39) | 0.433 | 1.15 (0.07, 2.71) | 1.15 (0.02, 2.63) | 1.26 (0.64, 2.56) | 0.86 |
| CRRT, n (%) | 31 (22) | 19 (15) | 12 (86) | < 0.001 | 10 (17) | 7 (13) | 3 (50) | 0.055 |
| Re-incubation, n (%) | 15 (11) | 10 (8) | 5 (36) | 0.008 | 4 (7) | 3 (6) | 1 (17) | 0.357 |
| Bentall, n (%) | 25 (18) | 20 (16) | 5 (36) | 0.132 | 10 (17) | 8 (15) | 2 (33) | 0.266 |
| Total-arch, n (%) | 117 (84) | 107 (85) | 10 (71) | 0.247 | 51 (86) | 46 (87) | 5 (83) | 1 |
| ICU, Median (Q1,Q3) | 7 (4, 14) | 7 (4, 13) | 12.5 (6, 19.25) | 0.218 | 6 (4, 13.5) | 6 (4, 11) | 9 (4, 20.75) | 0.588 |
| Hospitalization, Median (Q1,Q3) | 17 (13.75, 25) | 18 (14, 25) | 13 (6.25, 27) | 0.036 | 17 (13.5, 24.5) | 18 (14, 25) | 9 (4, 20.75) | 0.095 |

**Supplementary Table 1.** Baseline information of Training and Validation cohorts


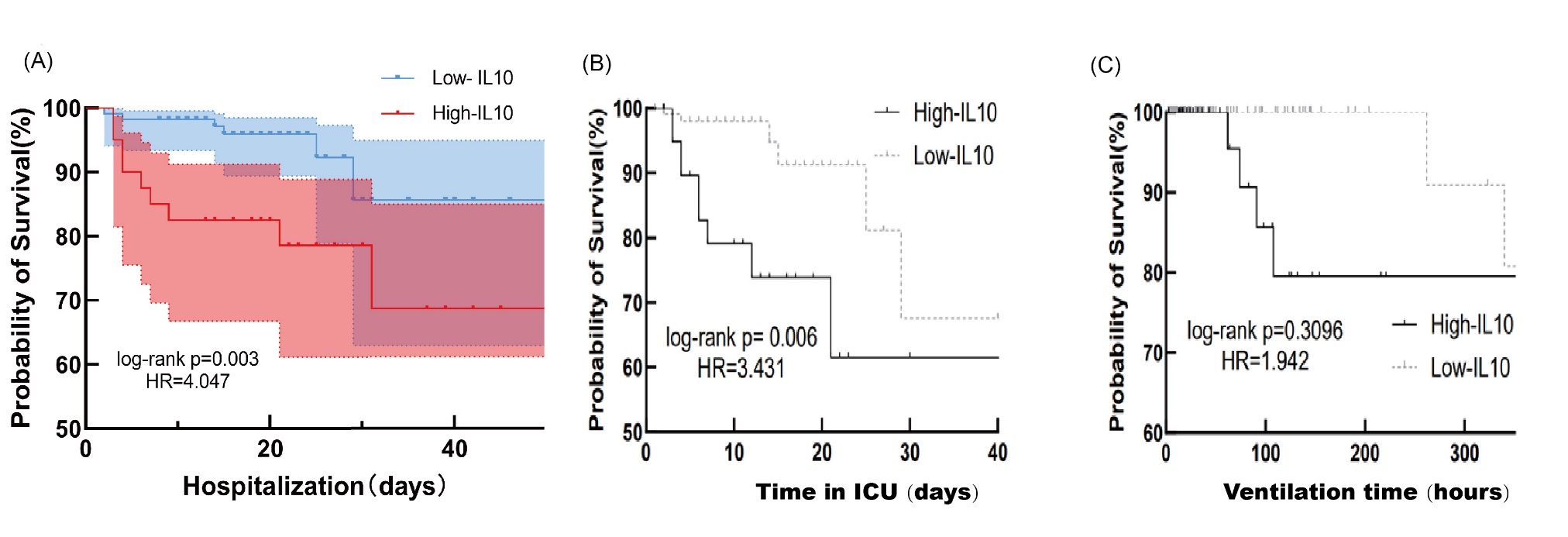


**Supplementary Fig. 1.** (A) In-hospital survival curve analysis based on patients with ATAAD (B and C) The Kaplan-Meier analysis for the time in ICU (A) and the time of Ventilation (B) after surgery.

HR: Hazard Ratio, Log-rank test: Mantel-Cox test; the color field covers the 95% CI of ratio.


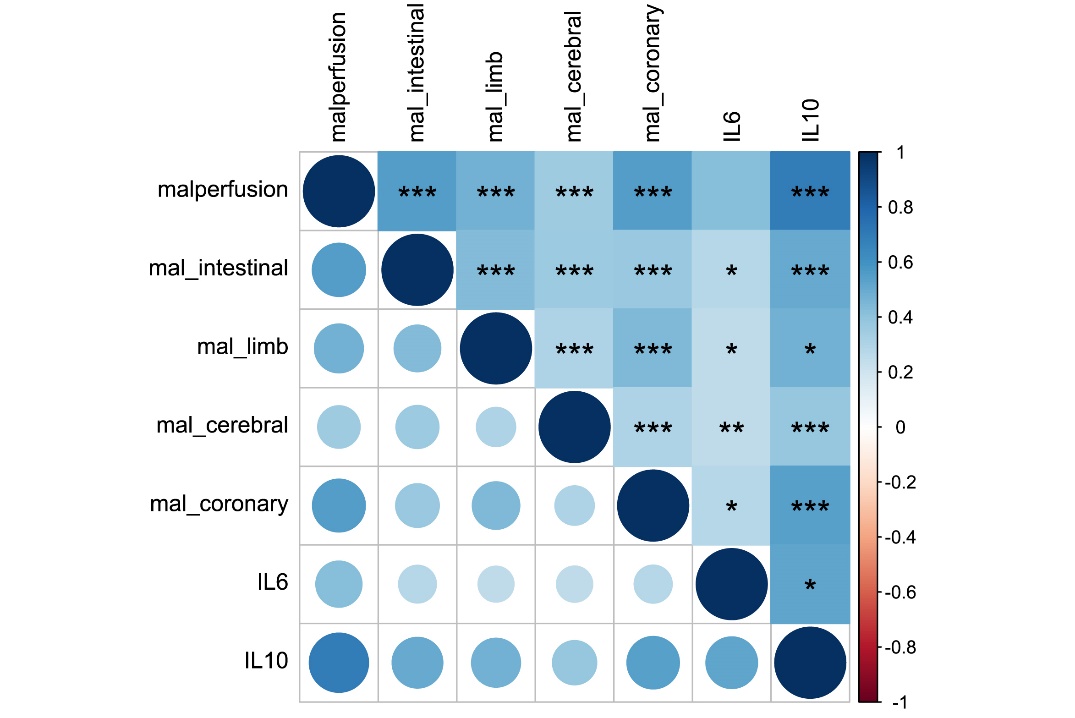


**Supplementary Fig. 2.** The association between IL-6 & 10 and end-organ mal-perfusion of TAAD patients. mal- : mal-perfusion of certain organ or system.
